# Response to: “Inhalation NO in the HFNC group may result in a meaningless extension of survival time”

**DOI:** 10.1186/s13054-024-05218-1

**Published:** 2025-01-07

**Authors:** Shahin Isha, Pablo Moreno Franco, Devang K. Sanghavi

**Affiliations:** Department of Critical Care Medicine, Mayo Clinic, 4500 San Pablo Rd S, Jacksonville, FL 32224 USA

Dear Editor,

We would like to thank Zhong et al. for the thoughtful comments regarding our manuscript “Impact of low-dose inhaled nitric oxide treatment in spontaneously breathing and intubated COVID-19 patients: a retrospective propensity-matched study” and welcome the opportunity to provide clarifications [1, 2]. While we acknowledge some of their points, others require further explanation to provide a clearer interpretation of our study findings.

First, Zhong et al. recommended that we use the initiation of inhaled nitric oxide (iNO) as the starting point for our survival analysis. As we can understand from the study design this was not a comparison between two interventions being provided but was rather a comparison between the intervention group (iNO) and standard of care group (HFNC/ intubation). Moreover, as Zhong et al. rightly pointed out, many times iNO was not started right away after HFNC initiation/ intubation. To establish a common timestamp for time-dependent survival analysis, we used the HFNC initiation time as the starting point for both the iNO and non-iNO groups. As the authors correctly pointed out, this approach can lead to immortal time bias. Therefore, we examined the exact dates of iNO initiation. We noted a median interval of 1 (IQR 0–4) days between HFNC initiation and the iNO start date. As suggested by the authors, we decided to perform additional analyses while considering iNO initiation and HFNC initiation as starting points for survival analyses for the iNO group and non-iNO group, respectively. Upon performing IPTW-weighted univariate Cox regression analysis, iNO use was found to have a lower risk of in-hospital mortality (HR: 0.48, 95% CI: 0.31–0.75, *p* = 0.001) as well as lower 28-day mortality risk (HR: 0.50, 95% CI: 0.31–0.79, *p* = 0.003).

The authors also inquired whether any adjustments for covariates were made in the Cox regression model. We would like to clarify that, since we used the propensity-matching and inverse propensity of treatment weighting (IPTW) approaches for our statistical analyses, we employed a weighted Cox univariate regression model for survival analysis. This method incorporates weights from multiple confounding variables, allowing us to estimate marginal hazard ratios and survival curves. This approach contrasts with traditional multivariate Cox models, which estimate conditional hazard ratios. These methodological choices are supported by prior published literature as well [3, 4]. Propensity weight was also taken into account to plot weighted Kaplan–Meier survival curves for visual representation (Figs. 3 and 4 in the original manuscript) [2].

We would also like to clarify that the mortality rates reported in Tables 1 and 4 of the original manuscript are in-hospital mortality and not 28-day mortality [2]. However, the Kaplan–Meier survival curves as demonstrated in Figs. 3 and 4 signify 30-day mortality risk. The discrepancy between crude in-hospital mortality rate difference and the hazard ratio derived from survival analysis has been noted in some prior literature and should be interpreted under appropriate contexts [5]. The hazard ratio obtained from Cox proportional hazard analysis reflects time-to-event dynamics, and it can be influenced by both time and event factors. The possibility of delaying mortality cannot be excluded and should ideally be explored through further large-scale prospective trials. We did, however, perform additional analyses to plot 90-day Kaplan–Meier survival curves as per the authors’ suggestion and have included that in this correspondence (Fig. 1). We had only four patients with a post-intervention length of stay (HFNC/ iNO start) of more than 90 days and therefore did not decide to include a 180-day survival curve as there was minimal difference compared to a 90-day survival curve.

**Fig. 1:**
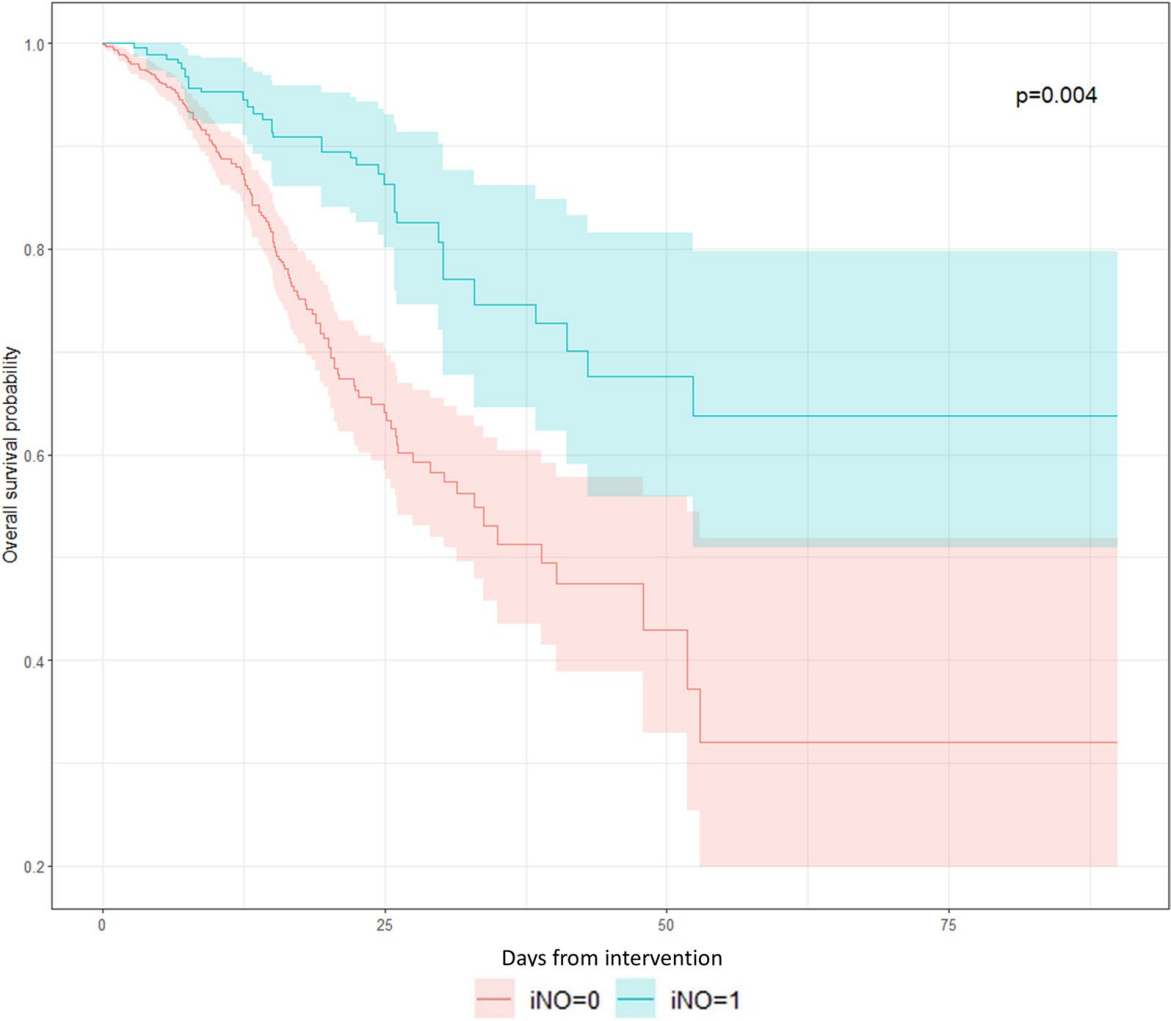
Weighted Kaplan–Meier survival curve demonstrating 90-day survival from the time since iNO initiation or HFNC initiation (for the non-iNO group)

We hope these clarifications address the concerns raised and further improve the clarity of our analytical approach as well as study findings. Thank you for allowing us to engage in this constructive discussion.

## Data Availability

Due to institutional policies, data is available upon reasonable request addressed to the corresponding author.

## Abbreviations

iNO: Inhaled nitric oxide
HFNC: High flow nasal cannula
COVID-19: Coronavirus disease 2019
HR: Hazard ratio
IPTW: Inverse propensity of treatment weighted
